# Association between Petal Form Variation and *CYC2*-like Genotype in a Hybrid Line of *Sinningia speciosa*

**DOI:** 10.3389/fpls.2017.00558

**Published:** 2017-04-18

**Authors:** Hao-Chun Hsu, Chun-Neng Wang, Chia-Hao Liang, Cheng-Chun Wang, Yan-Fu Kuo

**Affiliations:** ^1^Department of Bio-Industrial Mechatronics Engineering, National Taiwan UniversityTaipei, Taiwan; ^2^Institute of Ecology and Evolutionary Biology, National Taiwan UniversityTaipei, Taiwan; ^3^Department of Life Science, National Taiwan UniversityTaipei, Taiwan

**Keywords:** *CYCLOIDEA*, dorsoventral asymmetry, genotype–phenotype association, petal form variation, *Sinningia speciosa*, three-dimensional (3D) analysis, ventralization

## Abstract

This study used three-dimensional (3D) micro-computed tomography (μCT) imaging to examine petal form variation in a hybrid cross of *Sinningia speciosa* between a cultivar with actinomorphic flowers and a variety with zygomorphic flowers. The major objectives were to determine the genotype–phenotype associations between the petal form variation and *CYCLOIDEA2*-like alleles in *S*. *speciosa* (*SsCYC*) and to morphologically investigate the differences in petal types between actinomorphic and zygomorphic flowers. In this study, μCT was used to accurately acquire 3D floral images. Landmark-based geometric morphometrics (GM) was applied to evaluate the major form variations of the petals. Nine morphological traits of the petals were defined according to the form variations quantified through the GM analysis. The results indicated that the outward curvature of dorsal petals, the midrib asymmetry of lateral petals, and the dilation of ventral region of the tube were closely associated with the *SsCYC* genotype. Multiple analyses of form similarity between the petals suggested that the dorsal and ventral petals of actinomorphic plants resembled the ventral petals of zygomorphic plants. This observation indicated that the transition from zygomorphic to actinomorphic flowers in *S. speciosa* might be caused by the ventralization of the dorsal petals. We demonstrated that the 3D-GM approach can be used to determine genotype–phenotype associations and to provide morphological evidence for the transition of petal types between actinomorphic and zygomorphic flowers in *S. speciosa*.

## Introduction

*Sinningia* *speciosa* (Lodd.) Hiern is a species of ornamental plants with high diversity in floral form. The peloric form of the species, also known as peloric Gloxinia, has a floral form distinct from that of the wild progenitor variety (Citerne and Cronk, 1999; Zaitlin, 2012). Typically, the wild variety displays flowers with two internally asymmetric dorsal and lateral petals. The ventral petal is internally bilaterally symmetric; thus, the flower presents zygomorphic corollas. By contrast, the peloric Gloxinia displays flowers with all internally bilaterally symmetric petals. The transition between floral zygomorphy and actinomorphy is considered to result from the single gene effect of a homolog of the *CYCLOIDEA2*-like gene (*SsCYC*; Citerne et al., 2000; Hsu et al., 2015). Petal form is an important aspect in ornamental plant breeding. In this study, a wild variety and a peloric Gloxinia were crossed to breed a second-generation (F_2_) population. Three-dimensional (3D) micro-computed tomography (μCT) imaging and geometric morphometrics (GM; Lawing and Polly, 2010; Zelditch et al., 2012) techniques were applied to the F_2_ hybrid population to quantify the petal form variation. The associations between the petal form variation and the *SsCYC* allele were also evaluated.

The associations between the petal form variation and gene alleles have been investigated previously. Brock et al. (2012) used the natural populations of the model plant *Arabidopsis thaliana* to examine the association between petal form and genotypic variations. In addition, some studies in non-model plants, such as *Rhytidophyllum* (Alexandre et al., 2015), *Mimulus* (Fishman et al., 2015), and *Penstemon* (Wessinger et al., 2014), the quantitative trait loci (QTL) analysis was performed to determine the gene alleles that affect petal forms. In addition to the petal form variation in nature, the changes in petal form during floricultural domestication exhibited a strong link with a single gene (Chapman et al., 2012). In these studies, the petal form variation was evaluated through conventional morphometrics or were quantified using 2D images. Flowers, however, are 3D objects with complex geometries. The application of authentic 3D imaging to flowers is required to capture the form information implicit in the petals.

Modern imaging techniques have made the acquisition of 3D images of floral forms feasible and affordable. With the development and improvement of 3D imaging, X-ray μCT has been applied to plant materials in the last two decades (Stuppy et al., 2003; Fiorani and Schurr, 2013; Karahara et al., 2015). Recent studies combining 3D imaging with GM have analyzed form using the Cartesian coordinates of landmarks; this analysis method represents a powerful strategy to comprehensively capture the structural information of flowers and to precisely quantify floral form variations (van der Niet et al., 2010). Our previous work (Wang et al., 2015) proposed a procedure to capture floral images in 3D using μCT and to implement GM to quantify the floral shape variations. In the present study, we applied the 3D-GM approach to further examine floral form variations at the petal level for determining genotype–phenotype association and for testing the petal form similarity.

The petal form similarity between petal types in floral symmetry transition is an intriguing topic. Although floral symmetry changes across species or varieties, the transformation of petal type can be categorized into dorsalization and ventralization (Cronk, 2006). Dorsalization, in which the ventral petals resemble the dorsal petals, is observed in *Mohavea* (Hileman et al., 2003) and *Cadia* (Citerne et al., 2006). By contrast, ventralization, in which the dorsal petals resemble the ventral petals, is observed in *Primulina* (Yang et al., 2012) and *Lotus* (Feng et al., 2006). However, these studies determined the transformation of petal type by using naked eye examination and the coincidence with the expression patterns of *CYCLOIDEA2*-like genes. It is worth to note that the loss-of-function mutation of only one *CYCLOIDEA2*-like gene in *S. speciosa* might closely associate with floral symmetry transition (Citerne et al., 2000; Hsu et al., 2015). Taking advantage of the floral symmetry in peloric Gloxinia and the wild varieties, we aimed to observe and determine the transformation of petal type for *S. speciosa* geometrically by using the 3D-GM approach.

In this study, we aim to determine the association between 3D petal form variation and *SsCYC* genotypes, and to test the petal form similarity in a hybrid line with actinomorphic and zygomorphic flowers in *S. speciosa*. The specific objectives were to (1) define and quantify petal traits in the F_2_ population, (2) evaluate the association between the *SsCYC* genotype and petal traits, and (3) geometrically determine whether dorsalization or ventralization occurred in the F_2_ flowers.

## Materials and Methods

### Plant Materials and SsCYC Genotyping

The *S. speciosa* ‘Carangola’ with zygomorphic flowers and the *S. speciosa* ‘Peridots Darth Vaders’ with actinomorphic flowers were obtained from the Dr. Cecilia Koo Botanic Conservation and Environmental Protection Center (Pingtung, Taiwan). The ‘Peridots Darth Vaders’ was crossed by ‘Carangola’ to breed the first-generation (F_1_) plants in June, 2007. A single F_1_ plant was then selfed to cultivate the F_2_ populations in May, 2008. A total of 320 F_2_ plants were established. In the F_2_ population, one plant had four-lobe flowers, two plants had six- or seven-lobe flowers. The rest F_2_ plants had five-lobe flowers. The flowers of F_2_ plants that are not five-lobe were excluded because they were incomparable in form (Adams et al., 2004). In this study, 72 F_2_ plants were randomly selected for the experiment. The plants were grown in greenhouses under 22–28°C with 70–80% humidity and natural lighting with 20% shade.

The *SsCYC* nucleotide sequences of the two parental accessions and the F_2_ individuals were identified. See Hsu et al. (2015) for the details of the *SsCYC* genotyping. The *SsCYC* alleles of the zygomorphic and actinomorphic parents were denoted as *C* and *c*, respectively. The *SsCYC* genotypes of the hybrid line were then identified and denoted as *C/C*, *C/c*, and *c/c* for the homozygous zygomorphic alleles, heterozygous zygomorphic and actinomorphic alleles, and homozygous actinomorphic alleles, respectively. Among the 72 F_2_ plants, the ratio of *SsCYC* genotypic combinations (16, 43, and 13 individuals of genotypes *C*/*C*, *C*/*c*, and *c*/*c*, respectively) were tested under the null hypothesis with *C/C*:*C/c*:*c/c* = 1:2:1 at a significance level of 0.05.

### Image Acquisition and Landmark Selection

**FIGURE 1:**
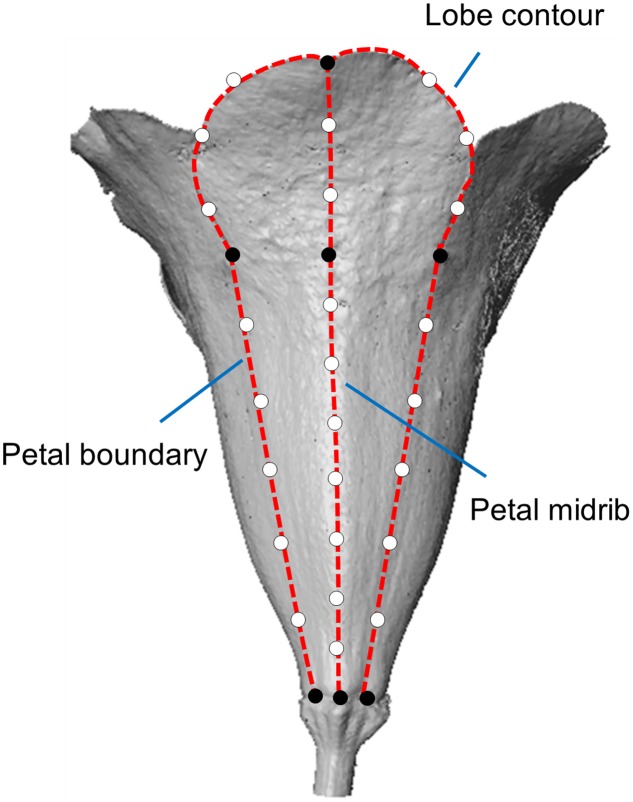
**The landmarks in a petal.** The black and hollow dots represent the primary and secondary landmarks, respectively. The dashed lines represent the lobe contour, petal boundaries, and petal midrib.

The flower images of the 72 plants were acquired during August 2012 and September 2015. See Wang et al. (2015) for details of the scanning process and image processing procedure. The strategy of landmark selection in the present study was different from the strategy in Wang et al. (2015). We added landmarks in petal boundary to describe the flower tube form more comprehensively (**Figure 1**). The landmark selection procedure was described as the follows. The primary landmarks were defined as the intersections of adjacent lobe contours, endpoints of the petal midrib, endpoints of the petal boundary, and joints of the tube and lobe on the midrib (black dots in **Figure 1**). The secondary landmarks were equally distributed between two primary landmarks (hollow dots in **Figure 1**). In the selection process, the petal features (e.g., lobe contours, petal midribs, and petal boundaries) and the primary landmarks were identified using an open source software program (Landmark; Wiley et al., 2005). The secondary landmarks were then determined using a program developed in MATLAB (The MathWorks, Natick, MA, USA). See Wang et al. (2015) for the details of the landmark selection. A total of 32 landmarks, comprising 7 primary and 25 secondary landmarks, were collected for each petal.

### Identification and Visualization of the Major Form Variations of the Petals

Geometric morphometrics was applied to the landmarks of the dorsal, lateral, and ventral petals to evaluate the between-flower form variations of the petals. See Wang et al. (2015) for details of the GM analysis. Thereafter, the major form variations of the petals were visualized. See Wang et al. (2015) for the details of the visualization.

### Morphological Traits of the Petals

**FIGURE 2:**
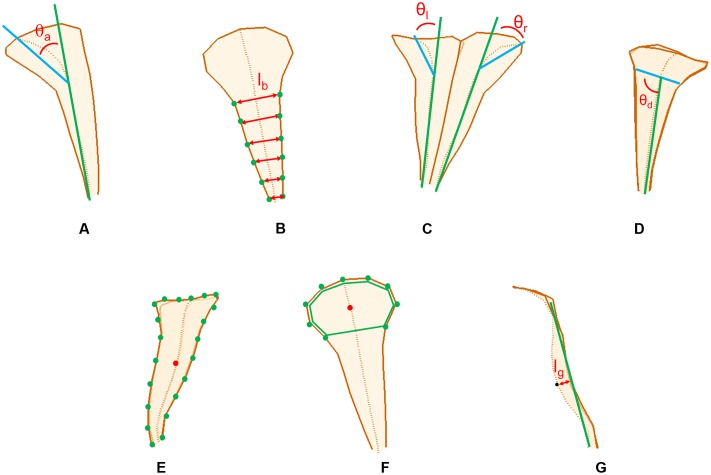
**The petal traits: (A)** petal outward curvature, **(B)** petal width, **(C)** left–right asymmetry, **(D)** midrib asymmetry, **(E)** petal size, **(F)** lobe size, and **(G)** tube dilation.

The morphological traits of the dorsal, lateral, and ventral petals were defined and quantified according to the principal form variations discovered using the GM analysis. The selection of these morphological traits were referenced from the description of floral morphologies in previous studies (Endress, 2001; Waites and Hudson, 2001; Nii and Kawabata, 2011; Mitteroecker et al., 2013; Hsu et al., 2015; Ding et al., 2016). The traits included petal outward curvature, petal width, left–right asymmetry, midrib asymmetry, petal size, lobe size, and tube dilation (**Figure 2**). The traits corresponding to these variations were then geometrically defined and directly quantified in the 3D flower images by using image-processing algorithms.

**FIGURE 3:**
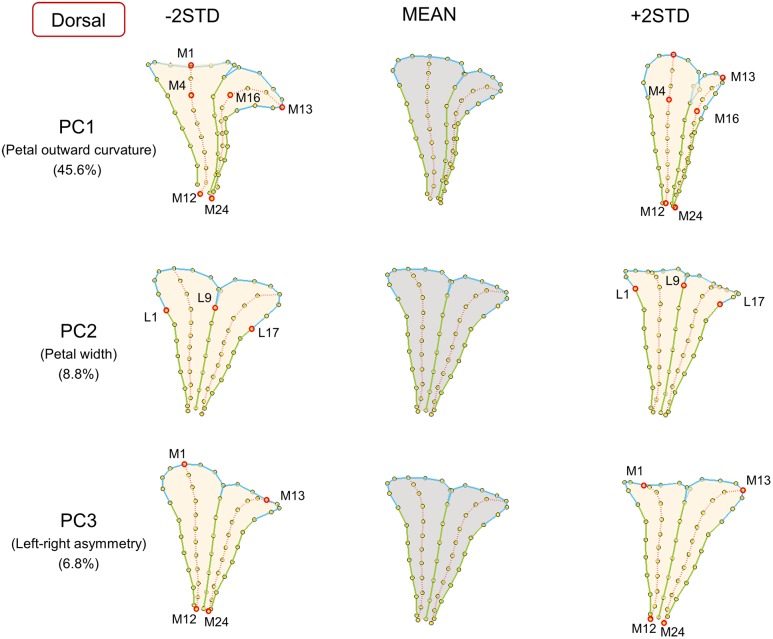
**The major shape variations of the dorsal petals.** The PC1, PC2, and PC3 scores accounted for 45.6, 8.8, and 6.8% of the total shape variation of dorsal petals, respectively. Yellow dots represent landmarks. Red circles represent labeled landmarks. Cyan, green, and red lines represent the lobe contours, tube boundaries, and petal midribs, respectively. The petal forms with mean PC values are illustrated in gray; the petal forms with PC values of mean ± 2 standard deviations are illustrated in beige.

**FIGURE 4:**
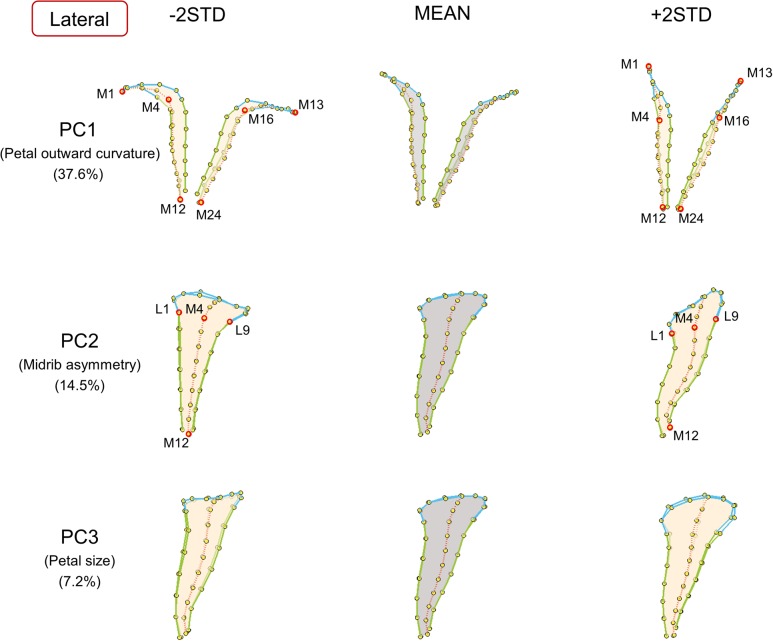
**The major shape variations of the lateral petals.** The PC1, PC2, and PC3 scores accounted for 37.6, 14.5, and 7.2% of the total shape variation of lateral petals, respectively. Yellow dots represent landmarks. Red circles represent labeled landmarks. Cyan, green, and red lines represent the lobe contours, tube boundaries, and petal midribs, respectively. The lateral petals are illustrated from top-view (PC1) or side-view (PC2 and PC3) to exhibit the shape variations. The petal forms with mean PC values are illustrated in gray; the petal forms with PC values of mean ± 2 standard deviations are illustrated in beige.

**FIGURE 5:**
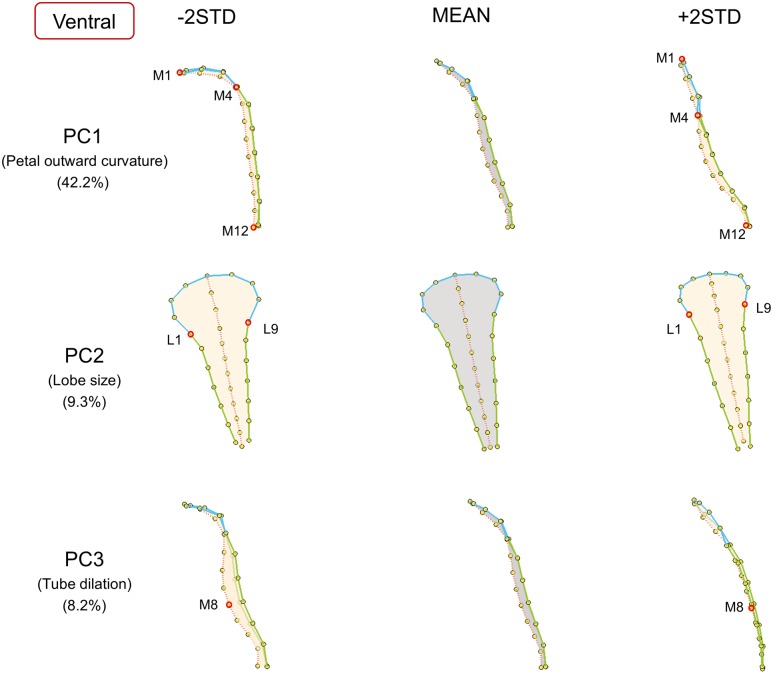
**The major shape variations of the ventral petals.** The PC1, PC2, and PC3 scores accounted for 42.2, 9.3, and 8.2% of the total shape variation of ventral petals, respectively. Yellow dots represent landmarks. Red circles represent labeled landmarks. Cyan, green, and red lines represent the lobe contours, tube boundaries, and petal midribs, respectively. The ventral petals are illustrated from side-view (PC1 and PC3) or front-view (PC2) to exhibit the shape variations. The petal forms with mean PC values are illustrated in gray; the petal forms with PC values of mean ± 2 standard deviations are illustrated in beige.

Petal outward curvature was defined as the angle (𝜃_a_ in **Figure 2A**) between the tube-growth line (the green line in **Figure 2A**) and lobe-bending line (the blue line in **Figure 2A**). The tube-growth line was defined as the line connecting the landmarks M4 and M12 in **Figures 3–5**. The lobe-bending line was defined as the line connecting the landmarks M1 and M4 in **Figures 3–5**. Petal outward curvature was quantified for the dorsal, lateral, and ventral petals. Petal width was defined as the mean of the distance (l_b_ in **Figure 2B**) between the corresponding landmarks on the petal boundaries (the green dots in **Figure 2B**). Petal width was quantified for the dorsal petals. Left–right asymmetry was defined as the difference between the outward curvature angles of the petals with the same petal type (𝜃_l_ and 𝜃_r_, respectively, in **Figure 2C**). Left–right asymmetry was quantified for the dorsal petals. Midrib asymmetry was defined as the angle (𝜃_d_ in **Figure 2D**) between the lobe–tube boundary (the blue line in **Figure 2D**) and tube-growth line (the green line in **Figure 2D**). The lobe–tube boundary was defined as the line connecting the landmarks L1 and L9 in **Figure 4**. Midrib asymmetry was quantified for the lateral petals. Petal size was defined as the centroid size of the petal (**Figure 2E**). The centroid size was the squared root of the summed squared distances between all landmarks (the green dots in **Figure 2E**) and their centroid (the red dot in **Figure 2E**). Petal size was quantified for the lateral petals. Lobe size was defined as the centroid size of the lobe (the green polygon in **Figure 2F**). Lobe size was quantified for the ventral petals. Tube dilation was defined as the distance (l_g_ in **Figure 2G**) from the midpoint of the tube midrib (the black dot in **Figure 2G**; landmark M8 in **Figure 5**) to the tube-growth line (the green line in **Figure 2G**). Tube dilation was quantified for the ventral petals. It was also referred to as ventral tube dilation. When a trait was quantified for the dorsal or lateral petals, each of which was composed of two petals, the mean value of the two petals was used.

Correlations between the defined traits and their corresponding principal form variations were examined. The purpose of the examination was to confirm whether the defined traits adequately represent the major form variations. The correlations among the defined traits were also investigated using correlation analysis. The trait difference between different *SsCYC* genotypes were tested using ANOVA and Scheffé’s multiple comparison test (Scheffé, 1953).

### Association between the SsCYC Genotypes and Traits

The logarithm of odds (LOD; Broman and Sen, 2009) scores between the *SsCYC* genotypes and the defined traits were calculated to provide evidence for the genotype–phenotype association. Permutation tests (Churchill and Doerge, 1994) were also performed to evaluate the levels of significance for the association. The null hypothesis of the permutation tests was that the *SsCYC* genotype was not associated with the traits. In the permutation tests, the statistic was the LOD scores. The null distribution was generated by reshuffling the phenotypes relative to the genotypes for 10,000 replicates. The *P*-value of a test was then determined as the frequency of the LOD scores that were equal to or greater than the original LOD score. The percentage of variance explained (PVE) was also calculated to determine the contribution of the *SsCYC* genotypes.

### Test of Petal Form Similarity

**FIGURE 6:**
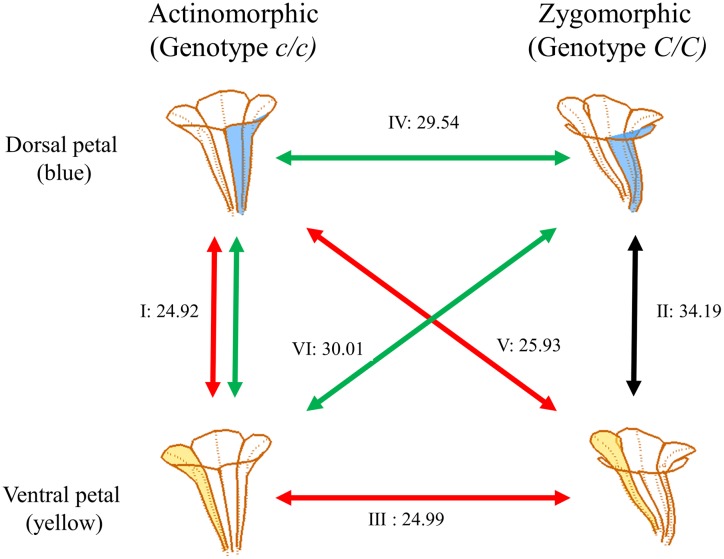
**Shape similarities between petals.** The dorsal and ventral petals are illustrated in blue and yellow, respectively. The numbers alongside the lines indicate the mean Hausdorff distances between the petal sets. The Roman numerals indicate the comparison of two-petal set pairs: (I) the dorsal and ventral petals of actinomorphic plants, (II) the dorsal and ventral petals of zygomorphic plants, (III) the ventral petals of actinomorphic and zygomorphic plants, (IV) the dorsal petals of actinomorphic and zygomorphic plants, (V) the dorsal petals of actinomorphic plants and the ventral petals of zygomorphic plants, and (VI) the ventral petals of actinomorphic plants and the dorsal petals of zygomorphic plants.

We morphologically examined (1) whether the dorsal petals of actinomorphic plants resemble the ventral petals of zygomorphic plants, rather than the dorsal petals of zygomorphic plants and (2) whether the ventral petals of actinomorphic plants resemble the dorsal petals of zygomorphic plants, rather than the ventral petals of zygomorphic plants. The examination was conducted by evaluating form similarity between two petals, each of which was selected from a petal set. The two-petal set pairs for comparison were (I) the dorsal and ventral petals of actinomorphic plants, (II) the dorsal and ventral petals of zygomorphic plants, (III) the ventral petals of actinomorphic and zygomorphic plants, (IV) the dorsal petals of actinomorphic and zygomorphic plants, (V) the dorsal petals of actinomorphic plants and ventral petals of zygomorphic plants, and (VI) the ventral petals of actinomorphic plants and dorsal petals of zygomorphic plants (**Figure 6**). Form similarities for all the combinations of the two-petal set pairs were examined, and their means were presented. Actinomorphic plants were assumed to have ventralized phenotypes if set pairs I, III, and V (red double arrows in **Figure 6**) had larger degrees of form similarities than did set IV. By contrast, actinomorphic plants were assumed to have dorsalized phenotypes if set pairs I, IV, and VI (green double arrows in **Figure 6**) had larger degrees of form similarities than did set III. Form similarity was evaluated using the Hausdorff distance (Huttenlocher et al., 1993) between the landmarks of the two petals. A small Hausdorff distance indicates a high degree of similarity between the two petals. After the Hausdorff distances were obtained, one-way ANOVA was performed to determine the difference in the Hausdorff distance among the petal sets. A Scheffé multiple comparison test was also conducted to determine which petal set tended to differ from the others. Because the plants of genotype *C*/*c* showed wide floral form variation, the individuals of genotype *C*/*c* were excluded from this examination.

## Results

### Major Form Variations of the Petals

**FIGURE 7:**
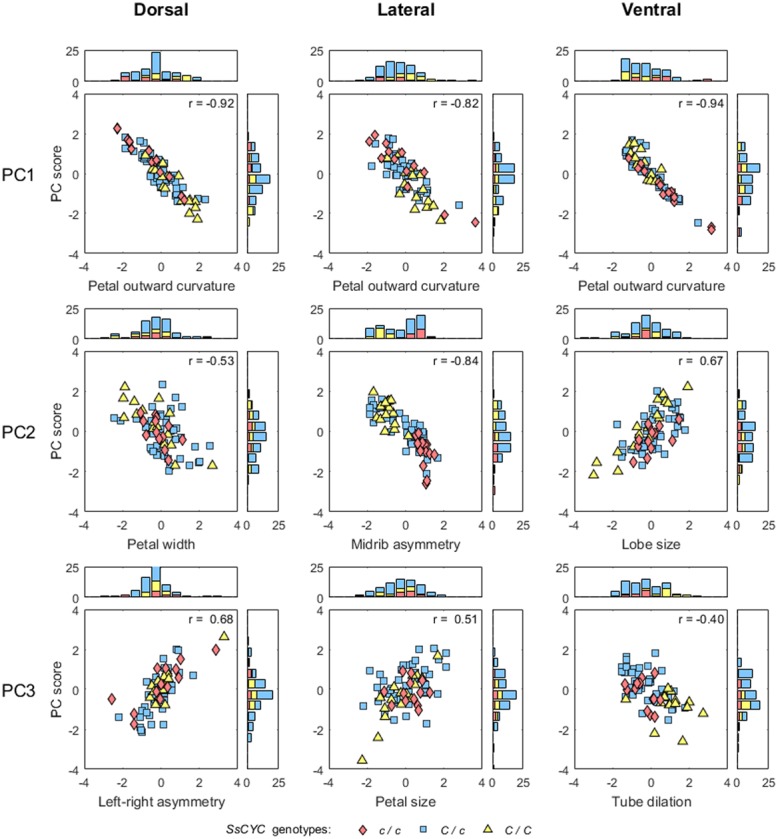
**Scatter plots and histograms of the PC scores and defined traits.** The PC scores and trait values are standardized. The correlation coefficients of the PC scores and trait values are provided at the upper right corners of the scatter plots.

Major petal form variation was identified using the proposed procedure. The first three principal components (PCs) accumulatively accounted for 61.2, 59.3, and 59.7% of the total form variations for the dorsal, lateral, and ventral petals, respectively. Each of the remaining PCs accounted for <5% of the total form variation. Thus, only the petal form variation corresponding to the first three PCs were presented. The PC scores were normally distributed (the right histograms in **Figure 7**). **Figures 3–5** visualize the major form variations of the dorsal, lateral, and ventral petals, respectively.

The major form variations of the dorsal, lateral, and ventral petals were examined. For the dorsal petals (**Figure 3**), we observed that PC1 was primarily associated with petal outward curvature. The lobes (the curves connecting landmarks M1–M4 and M13–M16) of the petals with small PC1 values bent outward at a considerable degree. By contrast, the lobes of the petals with large PC1 values were relatively straight. PC2 principally corresponded to the petal width. The widths (the distances between landmarks L1 and L9) of the petals with small PC2 values were greater than those of the petals with large PC2 values. PC3 was particularly related to left–right petal asymmetry. Among the petals with small PC3 values, the petal at one side remained straight, whereas the petal at the other side (the curves connecting landmarks M1–M12 and M13–M24) curved outward, and vice versa.

Among the lateral petals (**Figure 4**), PC1 principally corresponded to petal outward curvature. The lobes (the curves connecting landmarks M1–M4 and landmarks M13–M16) of the petals with small PC1 values bent outward at a substantial degree compared with those of the petals with large PC1 values. PC2 was primarily related to midrib asymmetry. The tube midribs (the curves connecting landmarks M4–M12) of the petals with large PC2 values were curved. By contrast, the tube midribs of the petals with small PC2 values were relatively straight. PC3 was particularly associated with petal size. Regarding size, the petals with small PC3 values were smaller than the petals with large PC3 values.

Among the ventral petals (**Figure 5**), PC1 primarily corresponded to petal outward curvature. The lobes (the curves connecting landmarks M1–M4) of the petals with small PC1 values bent outward at a significant degree, whereas the lobes of the petals with large PC1 values were relatively straight. PC2 was mainly related to lobe size. The lobe sizes (the area surrounded by landmarks L1–L9) of the petals with small PC2 values were larger than those of the petals with large PC2 values. PC3 was particularly associated with tube dilation. The tube centers (landmark M8) of the petals with small PC3 values protruded at a substantial degree. By contrast, the tubes of the petals with large PC3 values were relatively flat.

### Morphological Traits of the Petals

A correlation analysis was performed to determine whether the defined morphological traits adequately describe the form variations identified through GM analysis. The results revealed that the variation of the nine morphological traits was moderately or strongly correlated with their corresponding PC scores (**Figure 7**; absolute correlation coefficients = 0.40–0.94). The scores for these morphological traits were continuously distributed.

**FIGURE 8:**
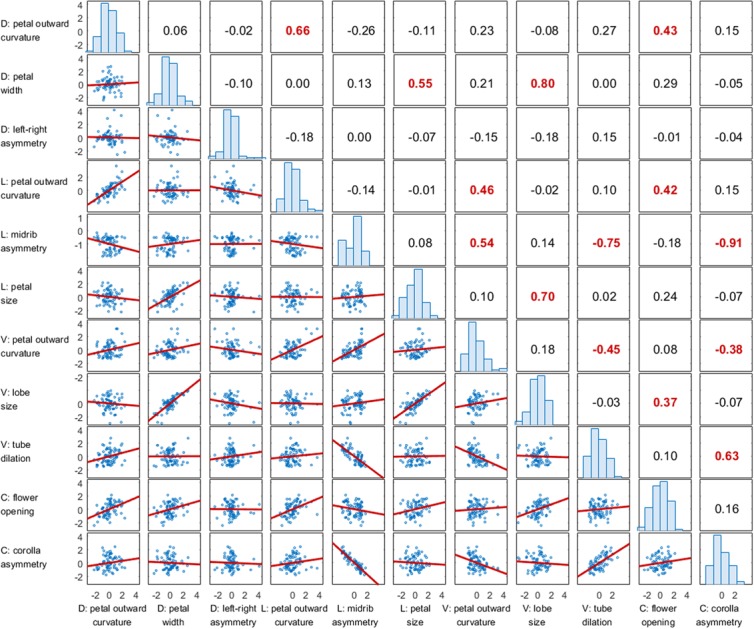
**Correlation matrix of the defined traits.** The numbers at the upper triangle indicate the correlation coefficients, and the *P*-values less than 0.005 are highlighted in red. The figures at the lower triangle indicate the correlation plots. The figures at the diagonal indicate the distributions of the defined traits. D, V, and L denote the dorsal, ventral, and lateral petals, respectively. C denotes the corolla.

The correlations among the morphological traits were also examined. Two additional traits, flower opening and corolla asymmetry, proposed by Wang et al. (2015) were included in the analysis. These two traits describe the form variation of the whole corolla versus the nine morphological traits defined in the present study, which were quantified specifically for individual petals. **Figure 8** shows the results of the pairwise correlation in a correlation matrix. The results revealed that the outward curvature of the dorsal and lateral petals were strongly correlated with each other (*r* = 0.66) and were also moderately correlated with flower opening (*r* = 0.42 and 0.43, respectively). This finding indicated that corollas with large degrees of flower opening led to large degrees of outward curvature of the dorsal and lateral petals. The petal size of the lateral petals, petal width of the dorsal petals, and lobe size of the ventral petals were moderately or strongly correlated with each other (*r* = 0.55, 0.70, and 0.80, respectively). Left–right asymmetry of the dorsal petals was not specifically correlated with any other trait (*r* < 0.20). Corolla asymmetry was strongly correlated with the midrib asymmetry of the lateral petals and the dilation of ventral region of the tube (*r* = -0.91 and 0.63, respectively). This observation indicated that a strong dorsoventral asymmetry was associated with the asymmetric midribs of the lateral petals and large ventral tube dilation.

**FIGURE 9:**
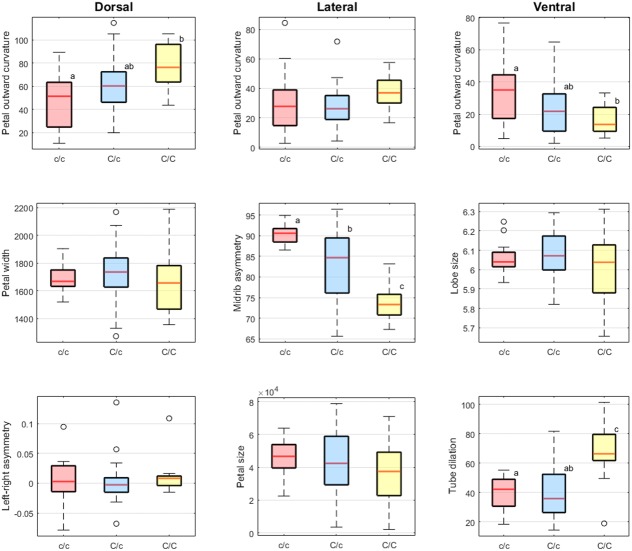
**Boxplots of the defined traits.** The lowercase alphabet near the box presenting the grouping result of Scheffé’s multiple comparison test.

**Table 1** summarizes the results of one-way ANOVA and multiple comparison analysis of the scores of defined traits versus *SsCYC* genotypes. One-way ANOVA revealed significant differences in the means of dorsal petal outward curvature, lateral midrib asymmetry, ventral petal outward curvature, and ventral tube dilation among the three genotypes (*F* > 5, *P* < 0.01). Results presented in **Figure 9** show that the scores of dorsal petal outward curvature and ventral tube dilation increased with the *c* allele replacing by the *C* allele. By contrast, the scores of lateral midrib asymmetry and ventral petal outward curvature decreased with the *c* allele replacing by the *C* allele. This observation implies that the *SsCYC* genotype may be associated with dorsal petal outward curvature, lateral midrib asymmetry, ventral petal outward curvature, and ventral tube dilation.

**Table 1:** ANOVA and Scheffé’s multiple comparison test of the petal traits.

Petal type	Trait	ANOVA		Scheffé’s multiple comparison test					
		*F*-value	*P*-value	*c/c* vs *C/c*		*c/c* vs *C/C*		*C/c* vs *C/C*	
				*T*-value	*P*-value	*T*-value	*P*-value	*T*-value	*P*-value
**Dorsal**	Petal outward curvature	7.620	0.001	1.853	0.187	3.815	0.001	2.861	0.021
	Petal width	1.741	0.183	0.964	0.631	0.586	0.843	1.788	0.209
	Left-right asymmetry	0.467	0.629	0.292	0.958	0.509	0.879	0.965	0.630
**Lateral**	Petal outward curvature	2.526	0.087	0.498	0.884	1.340	0.412	2.247	0.088
	Midrib asymmetry	21.057	<0.001	3.590	0.003	6.441	<0.001	4.332	<0.001
	Petal size	1.074	0.347	0.665	0.802	1.424	0.368	1.097	0.550
**Ventral**	Petal outward curvature	5.904	0.004	2.631	0.037	3.391	0.005	1.479	0.341
	Lobe size	1.792	0.174	0.337	0.945	1.195	0.493	1.888	0.176
	Tube dilation	17.486	<0.001	0.131	0.992	4.574	<0.001	5.690	<0.001


### Association between the SsCYC Genotypes and Defined Traits

**Table 2** summarizes the LOD scores, *P*-values from the permutation tests, and PVE. The genotype–phenotype association analysis revealed that the dorsal petal outward curvature, the lateral petal midrib asymmetry, and the ventral tube dilation were closely associated with the *SsCYC* genotypes (LOD > 3; *P* < 0.001; PVE > 15%). In addition, the *SsCYC* genotypes were strongly associated with corolla asymmetry (LOD = 6.22; *P* < 0.001; PVE = 32.8%).

**Table 2:** Logarithm of odds scores of the petal and corolla traits.

Petal type	Trait	LOD score	*P*-value	PVE (%)
**Dorsal**	Petal outward curvature	3.12	<0.001	18.2
	Petal width	0.77	0.178	1.3
	Left-right asymmetry	0.21	0.638	<0.1
**Lateral**	Petal outward curvature	1.10	0.087	3.1
	Midrib asymmetry	7.45	<0.001	38.7
	Petal size	0.48	0.346	3.0
**Ventral**	Petal outward curvature	2.47	0.004	14.5
	Lobe size	0.79	0.179	2.8
	Tube dilation	6.41	<0.001	23.3
**Corolla**	Flower opening	1.11	0.086	5.2
	Corolla asymmetry	6.22	<0.001	32.8


### Ventralization of the Dorsal Petals on the Actinomorphic Plants

**FIGURE 10:**
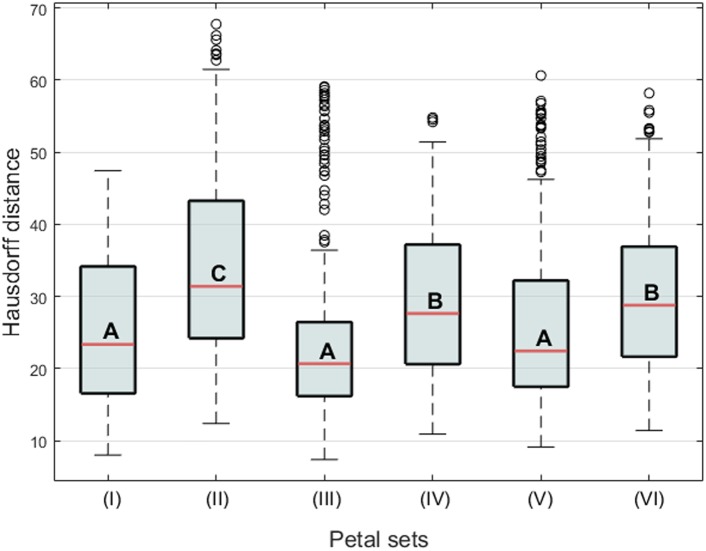
**Boxplot of the Hausdorff distances between the petal sets.** The letters A, B, and C indicate the groups of the petals. The Roman numerals indicate the comparison of two-petal set pairs, (I) the dorsal and ventral petals of actinomorphic plants, (II) the dorsal and ventral petals of zygomorphic plants, (III) the ventral petals of actinomorphic and zygomorphic plants, (IV) the dorsal petals of actinomorphic and zygomorphic plants, (V) the dorsal petals of actinomorphic plants and the ventral petals of zygomorphic plants, and (VI) the ventral petals of actinomorphic plants and the dorsal petals of zygomorphic plants.

Form similarities between the petal sets were evaluated. The mean Hausdorff distances between the dorsal and ventral petals of actinomorphic plants (set pair I), dorsal and ventral petals of zygomorphic plants (set pair II), ventral petals of actinomorphic and zygomorphic plants (set pair III), dorsal petals of actinomorphic and zygomorphic plants (set pair IV), dorsal petals of actinomorphic plants and ventral petals of zygomorphic plants (set pair V), and ventral petals of actinomorphic plants and dorsal petals of zygomorphic plants (set pair VI) were 24.92, 34.19, 24.99, 29.54, 25.93, and 30.01, respectively (**Figure 6**). One-way ANOVA revealed significant differences in the mean Hausdorff distances among the six set pairs (*F* = 20.03, *P* < 0.001). In the multiple comparison test, no significant difference was observed in the means of the Hausdorff distances of the set pairs I, III, and V (Group A in **Figure 10**). By contrast, the Hausdorff distances of these three set pairs were significantly lower than those of the set pairs II, IV, and VI (Groups B and C in **Figure 10**). The results indicated that the petals in the set pairs I, III, and V (red double arrows in **Figure 6**) resembled each other more closely than the petals in the set pairs IV and VI (green double arrows in **Figure 6**). This evidence may support the implication that the change of *SsCYC* allele combinations from *C*/*C* to *c*/*c*, which reflects the transition from zygomorphic to actinomorphic flowers, resulted in the ventralization of the dorsal petals.

## Discussion

In this study, 3D-GM was applied to *S. speciosa* to determine the petal form variation. The petal traits were then defined according to the analysis of the form variations of each petal. We evaluated the association of the variation of the defined traits with the *SsCYC* genotypes. We also provided morphological evidence suggesting that ventralization contributes to the development of the dorsal petals in peloric Gloxinia.

### 3D Technology Improving the Phenotyping of Petal Forms

Defining appropriate traits is a crucial challenge for plant phenotyping and genotype–phenotype association analysis (Fiorani and Schurr, 2013). In this study, the petal traits were defined by observing the 3D visualized petal form variation and were justified by conducting correlation analyses between the traits and form variations. The moderate-to-high correlation coefficients suggested that these defined petal traits accurately and sufficiently described the major form variations of the petals. In GM analysis, only one trait was defined from each PC. Taking PC1 of the dorsal petal as an example, the petal outward curvature accounted for the most significant change in the visualized form variation (**Figure 3**). A high correlation coefficient was observed between the variation of dorsal petal PC1 and the variation of petal outward curvature (*r* = -0.92). Nevertheless, some minor variations may exist, such as the lobe size variation observed in the visualized form variations for PC1 of the dorsal petal. The correlation analysis was sufficient to support that the defined petal traits were valid for describing the petal form variation for genotype–phenotype association analysis.

### Association between the SsCYC Genotypes and Defined Traits

The petal outward curvature of the dorsal petals, midrib asymmetry of the lateral petals, and the ventral tube dilation were associated with the *SsCYC* genotypes. This finding suggested that *SsCYC* is globally involved in floral form variation. The *CYCLOIDEA2*-like homolog genes play a role in determining the development of floral zygomorphy in many flowering plant lineages (Citerne et al., 2010; Hileman, 2014). According to most floral symmetry studies, *CYCLOIDEA2*-like genes mainly affect the development of the dorsal petals. However, our genotype–phenotype association analysis provided evidence that the phenotypical effects of the *SsCYC* alleles differ across the dorsal, lateral, and ventral petals. Particularly, the *SsCYC* genotypes were closely associated with the midrib asymmetry of the lateral petals and the ventral tube dilation (**Figure 9** and **Table 2**). Thus, the *SsCYC* in *S. speciosa* may have different effects in the shaping of different petals.

The *SsCYC* allele *C* may have different genetic effects on the petal traits. Based on the results presented in **Figure 9**, the *SsCYC* allele *C* seems to have additive genetic effect in the petal outward curvature of the dorsal petals and midrib asymmetry of the lateral petals. Although the LOD score of ventral petal outward curvature does not reach the significant criteria, it is worth to note that the *SsCYC* allele *C* has opposite effects in petal outward curvature of the dorsal and ventral petals. Particularly, the *SsCYC* allele *C* presents recessive genetic effect in the ventral tube dilation. These evidence might suggest that the *SsCYC* allele *C* leads the individuals of genotype *C/C* to have a specific floral form.

*CYC* regulates downstream genes, such as *RADIALIS* (*RAD*) and *DIVARICATA* (*DIV*), for shaping the morphology of the lateral and ventral petals in *Antirrhinum majus* (Almeida et al., 1997; Galego and Almeida, 2002). *CYC* and these downstream genes form an exclusive signaling network for establishing floral zygomorphy (Corley et al., 2005; Raimundo et al., 2013). Therefore, the effects of *RAD* and *DIV* on flower forms in *S. speciosa* should be investigated. However, only few studies have reported *RAD*–*DIV* interactions across certain Lamiales, Gesneriaceae, and Dipsacales species, and whether this interaction is conserved in *S. speciosa* remains to be further confirmed (Zhou et al., 2008; Preston et al., 2009; Boyden et al., 2012). Because *CYC* is epistatic to *RAD*, the *CYC* phenotypic changes may be included in the phenotypic effect of these downstream genes on floral symmetry. Thus, our analysis based on *CYC* captured the major form changes along the floral symmetry transitions. Additional studies validating the association between these genes and petal traits may extend our understanding regarding whether these genes contribute greatly to petal forms changes.

### Ventralization of the Dorsal Petals in Peloric Gloxinia

The evolution of floral symmetry has long been an intriguing topic for plant evolutionary-developmental (evo-devo) biologists. In flowering plant phylogeny, floral zygomorphy has been independently derived from actinomorphic ancestors many times (Citerne et al., 2010; Reyes et al., 2016). To elucidate the origin of floral zygomorphy, the concepts of dorsalization and ventralization were proposed for summarizing the petal type transition (Almeida et al., 1997; Cronk, 2006). Our petal form similarity analysis provided morphological evidence that the floral actinomorphy of peloric Gloxinia may result from the ventralization of the dorsal petals. This transition was also strongly associated with the genotype *c/c* in the *SsCYC* gene. According to floral symmetry studies in *Antirrhinum* (Luo et al., 1996; Almeida et al., 1997), the inactivated *CYC* mutant exhibited ventralization in the dorsal petals. Our finding would suggest that the *SsCYC* allele *c* serves as a loss-of-function mutation, causing the reversal of floral actinomorphy from floral zygomorphy in peloric Gloxinia.

## Concluding Remarks

In this study, the 3D quantification of the petal form variation was conducted in the F_2_ plants of *S. speciosa*. The morphological traits of petal (e.g., petal outward curvature, petal width, left–right asymmetry, petal size, midrib asymmetry, lobe size, and tube dilation) were defined based on the major form variations discovered through GM. Genotype–phenotype association analysis revealed that the petal outward curvature of the dorsal petals, midrib asymmetry of the lateral petals, and the dilation of ventral region of the tube were associated with the *SsCYC* genotypes. This finding suggested that the *SsCYC* gene plays different roles in the dorsal, lateral, and ventral petals and contributes to the integration of floral development. The form similarity analysis in petals suggested that the floral form transition between peloric Gloxinia and its wild varieties resulted from the ventralization of the dorsal petals.

## Author Contributions

The experiments were designed by Y-FK. The flower material was prepared by H-CH and C-NW. The experiments were performed by C-HL and C-CW. The data were processed, analyzed, and interpreted by C-HL, C-CW, H-CH, and Y-FK. The manuscript was prepared by H-CH, C-NW, C-HL, and Y-FK.

## Conflict of Interest Statement

The authors declare that the research was conducted in the absence of any commercial or financial relationships that could be construed as a potential conflict of interest.
